# Functional Validation of Noncoding Variants Associated With Nonsyndromic Orofacial Cleft

**DOI:** 10.1155/humu/6824122

**Published:** 2025-08-28

**Authors:** Siying Zhu, Hongxu Tao, Robert A. Cornell, Huan Liu

**Affiliations:** ^1^State Key Laboratory of Oral & Maxillofacial Reconstruction and Regeneration, Key Laboratory of Oral Biomedicine Ministry of Education, Hubei Key Laboratory of Stomatology, School & Hospital of Stomatology, Wuhan University, Wuhan, China; ^2^Department of Oral Health Sciences, School of Dentistry, University of Washington, Seattle, Washington, USA; ^3^Frontier Science Center for Immunology and Metabolism, Wuhan University, Wuhan, China; ^4^Taikang Center for Life and Medical Sciences, Wuhan University, Wuhan, China

**Keywords:** cisregulatory element, genome-wide association study, noncoding variant, nonsyndromic orofacial cleft

## Abstract

Over the past decade, genome-wide association studies (GWASs) have found genetic variants associated with elevated risk for nonsyndromic orofacial cleft (NSOFC). In the post-GWAS era of NSOFC genetic research, an important aim is to identify the pathogenic variants that influence craniofacial development processes, towards understanding how they lead to disease manifestation. However, two major challenges hinder the translation of GWAS results into a mechanistic understanding. Firstly, it is uncertain whether the variants pinpointed by GWAS represent the underlying pathogenic variants; secondly, the bulk of genetic variants identified through GWAS are situated in noncoding regions of the genome, complicating their biological interpretation. Presently, research on noncoding genetic variants associated with NSOFC predominantly centers on variants located in transcriptional regulatory elements. These variants modulate transcription, subsequently altering the expression of downstream target genes and disrupting gene regulatory networks. We provide a systematic summary of the recent NSOFC-associated GWAS findings for the first time. With a particular focus on variants located in noncoding regions, we delve into current statistical methods and functional approaches for identifying and validating causal variants, aiming to bridge the gap between genetic variants identified by GWAS and their underlying pathogenic mechanism responsible for NSOFC. Deciphering causal variants underlying NSOFC offers valuable clinical insights that may advance early diagnosis, enhance risk stratification, and facilitate the discovery of novel therapeutic targets.

## 1. Introduction

Orofacial cleft (OFC) is among the most prevalent complex genetic disorders, with a global incidence of about 1 per 700 live births, with considerable variation among ethnic groups [1]. Approximately 70% of OFC cases, devoid of other developmental anomalies, fall under the category of nonsyndromic orofacial cleft (NSOFC), attributed to the involvement of multiple genes and several environmental risk factors [2, 3]. NSOFC is frequently classified into two subtypes: nonsyndromic cleft palate only (NSCPO) and nonsyndromic cleft lip with or without cleft palate (NSCL/P) which includes nonsyndromic cleft lip only (NSCLO) and nonsyndromic cleft lip with cleft palate (NSCLP). Studies on twins and familial clustering have highlighted the significant genetic contributions to the etiology of NSOFC [4]. To date, a large number of susceptibility genes/loci achieving genome-wide significance have been identified through 15 independent genome-wide association studies (GWASs) [5–19], along with numerous subsequent meta-analyses and replication studies [20–36].

Despite the notable achievements of GWAS, the challenge of explaining the missing heritability and pinpointing causal variants persists. GWAS can account for approximately two-thirds of the heritability [37, 38]. One explanation for the missing heritability is the inadvertent exclusion of a myriad of common variants with marginal effect and rare variants failing to meet the stringent genome-wide significance threshold (typically set at *p* < 5 × 10^−8^) [39–41]. The quest for the missing fraction often necessitates sequencing of whole-genome or targeted genomic regions [42]. However, the precise identification of functional variants poses a formidable challenge. Owing to the phenomenon of linkage disequilibrium (LD), the lead single nucleotide polymorphisms (SNPs) initially highlighted by GWAS may not be causal variants within a haplotype block, with pathogenic variants equally prone to being in strong LD with them [41]. Furthermore, the bulk of SNPs associated with diseases uncovered by GWAS or sequencing are situated in noncoding regions of the genome, rendering their biological interpretation inherently challenging [39]. One subset of these SNPs is hypothesized to modulate the function of noncoding RNA (ncRNA) species (such as long ncRNA and microRNA [miRNA]) [43, 44] and another to affect cisregulatory elements (CREs) including enhancers, inhibitors, boundary elements, and promoters [45]. Consequently, diverse strategies encompassing statistical fine-mapping and expression quantitative trait locus (eQTL) analyses have been employed to sift through significant variants unearthed by GWAS in search of putative functional variants [46, 47]. While efforts have been made to prioritize candidate variants within noncoding regions, discerning the identity of downstream genes modulated by them remains elusive. In addition, the interplay between variants and ncRNA remains poorly understood. CREs can be present at varying distances from the genes they regulate [48]. Furthermore, a single CRE may exert regulatory influence over multiple target genes [49, 50]. Moreover, promoters and enhancers sometimes overlap, and variants may concurrently influence the activity of both [51]. These observations underscore the need for functional experimentation to unravel the mechanisms through which alterations in noncoding sequences impact the activity of CREs and expression or function of targeted genes.

Here, we first provided a synopsis of noncoding variants associated with NSOFC identified through GWAS and sequencing. Next, we summarized strategies for prioritizing candidate variants, including fine-mapping variants, analyzing eQTL results, sequencing genomes at high-throughput, primarily focusing on the identification of CREs, and applying machine learning techniques. Lastly, we scrutinized the current experimental pipelines used for validating the functional significance of noncoding variants.

## 2. Known Noncoding Variants Associated With NSOFC

**Figure 1:**
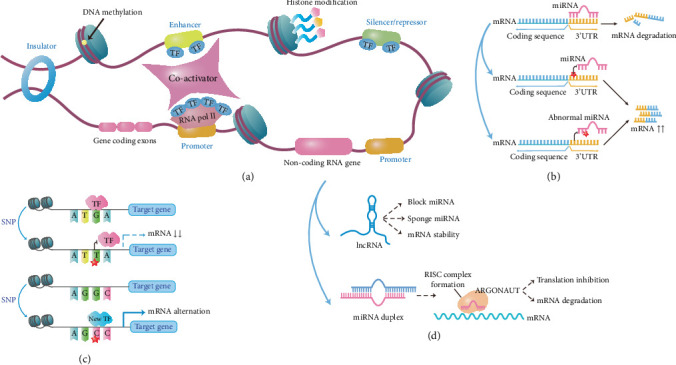
Impact of noncoding variants within cisregulatory elements (CREs) on gene regulation and disease pathogenesis. (a) Schematic representation of major regulatory elements in the genome, including promoters, enhancers, silencers/repressors, insulators, and noncoding RNA genes. These regions recruit TFs and cofactors, undergo epigenetic regulation (e.g., DNA methylation and histone modification), and coordinate gene expression [45]. (b) Effect of miRNA variants on posttranscriptional regulation. Canonical binding of a miRNA to the 3⁣′UTR of a target mRNA leads to mRNA degradation (top panel). Variants within the 3⁣′UTR (middle panel) or the miRNA seed region (bottom panel) can disrupt miRNA–mRNA interaction, thereby reducing repression efficiency and increasing mRNA expression [52]. (c) Effects of noncoding variants on TF binding. SNPs within CREs can disrupt existing TF binding sites, altering (elevating or reducing) mRNA expression (upper panel), or create novel TF binding sites, leading to altered regulation (lower panel) [45]. (d) Mechanisms of noncoding RNA–mediated regulation. lncRNAs regulate gene expression by blocking or sponging miRNAs, or by stabilizing target mRNAs (top panel). miRNAs are processed into duplexes and incorporated into the RNA-induced silencing complex (RISC) with ARGONAUT proteins to mediate translational repression or mRNA degradation (bottom panel) [52]. The figure was generated and refined in Adobe Illustrator. TF, transcription factor; 3⁣′UTR, 3⁣′ untranslated region; SNP, single nucleotide polymorphism; lncRNA, long noncoding RNA; miRNA, microRNA; RISC, RNA-induced silencing complex. Red star indicates the location of an SNP.

**Figure 2:**
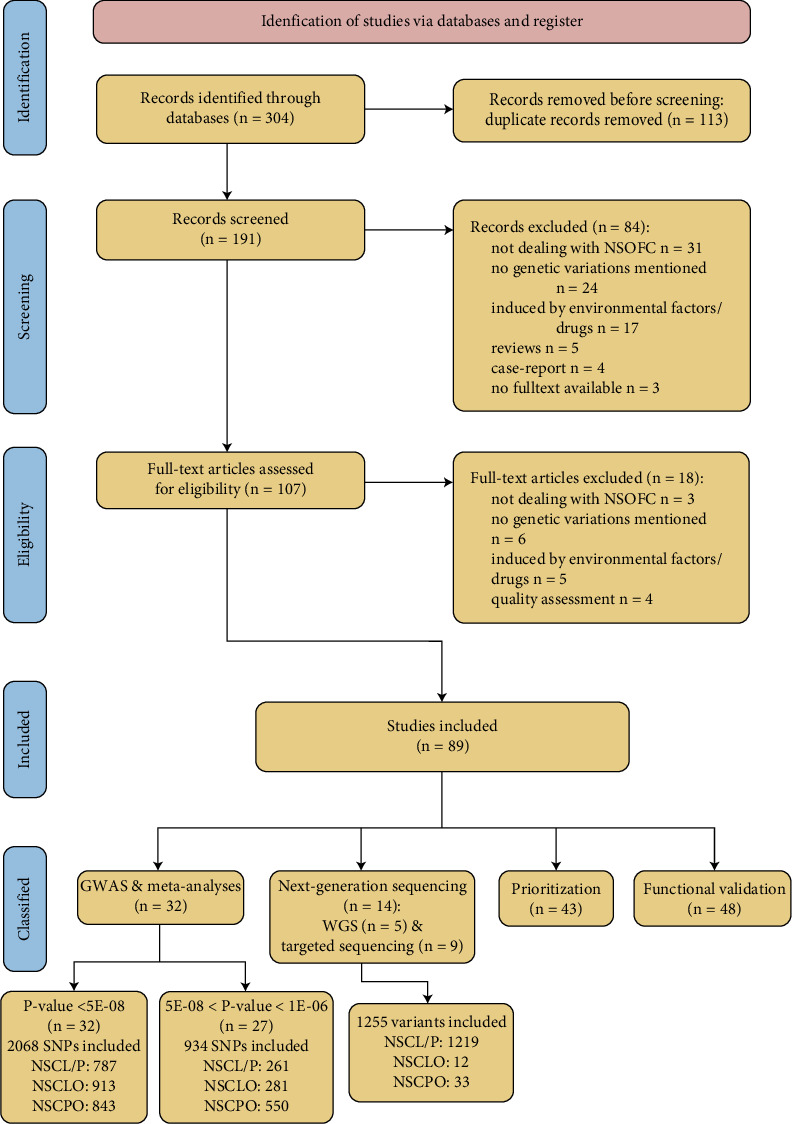
Schematic flowchart illustrating criteria for study inclusion. Flow diagram illustrating the systematic literature search strategy, beginning with a broad MEDLINE search via PubMed to identify all potentially relevant articles. Text mining of article titles, abstracts, and metadata was used to narrow down the initial results, leading to the exclusion of 84 articles. A total of 107 articles passed the initial screening and were manually reviewed for eligibility. The final systematic review included 89 articles, which were classified into four major categories: GWAS and meta-analyses, next-generation sequencing (including WGS and targeted sequencing), variant prioritization, and functional validation. Reasons for exclusion at each stage are shown on the right.

Noncoding regions encompass introns, CREs, 5⁣′ and 3⁣′ untranslated regions (UTRs) of mRNAs, and a range of ncRNAs, and variation in any of these elements may influence disease pathogenesis (Figure 1). miRNAs are a subset of ncRNAs, and at least one miRNA-140 regulates palatogenesis [53]. The UTRs and introns collectively constitute approximately 35% of the human genome [54]. Mutation of UTRs bordering the coding sequence of mRNA can affect the binding of miRNAs and RNA-binding proteins, contributing to the development of congenital diseases [52]. The introns are removed during pre-mRNA splicing, and abnormalities in this process can result in inherited diseases [55]. CREs include enhancers, promoters, silencers, and insulators. The bulk of this review will focus on how variation within CREs, identified in GWAS and high-throughput sequencing studies, can alter their interaction with transcription factors (TFs), thereby modulating the transcription of NSOFC-related genes [16, 45]. From the burgeoning field, we selected 87 articles related to NSOFC-associated noncoding variants for this review; our efforts were certainly incomplete, and additional studies are mentioned in the Supporting Information. Our literature screening process has been illustrated in Figure 2.

### 2.1. GWAS on NSOFC

**Figure 3:**
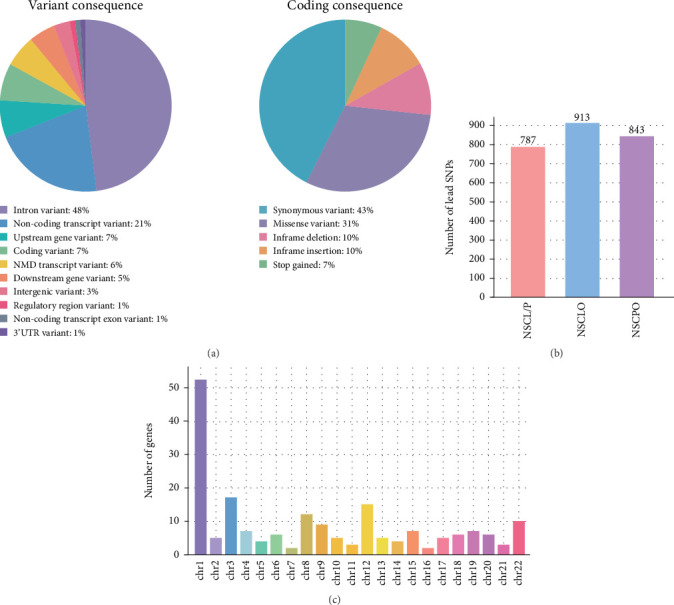
Summary of lead SNPs identified by GWAS. (a) Genomic annotation of significant variants associated with NSOFC identified by GWAS using the Ensembl Variant Effect Predictor [56]. (b) Number of lead SNPs identified by GWAS and meta-analyses in NSCL/P, NSCLO, and NSCPO, respectively. (c) Number of genes (192 in total) proximal to the lead SNPs across chromosomes. The figure was initially generated using the Ensembl Variant Effect Predictor and GraphPad Prism (based on data from Table S1) and further refined in Adobe Illustrator.

Since the first GWAS associated with NSCL/P was conducted by Birnbaum et al. in 2009 [5], there have been 32 NSOFC-associated GWAS and whole-genome meta-analyses; together, these studies identified 2068 lead SNPs achieving whole-genome significance (*p* value < 5e − 08) associated with NSOFC, proximal to 192 genes in the genome (Table S1, Figure 3) [5–36]. Associations were considered genome-wide significant if they reached a *p* value ≤ 5e − 08, the commonly accepted threshold based on Bonferroni correction for multiple testing [57]. Through genomic annotation by the Ensembl Variant Effect Predictor [56], approximately 7% of these SNPs are located in coding sequence (Figure 3a), a similar fraction as the SNPs associated with cardiovascular disease [58]. NSOFC-associated SNPs are distributed in the different categories of noncoding regions: introns (48%), ncRNAs (28%), flanking regions (upstream and downstream, 12%), intergenic regions (3%), UTRs (1%), and CREs (~1%) (Figure 3a). Interestingly, among SNPs in CREs, about 88% are in CREs active in specific tissues, as opposed to active in all cell types [59].

It is important to recognize that NSOFC comprises several phenotypes with overlapping but distinct genetic underpinnings. While early GWAS predominantly targeted NSCL/P, subsequent research has progressively examined other phenotypes. In 2016, Leslie et al. pioneered a GWAS of NSCPO and pinpointed a missense variant in *GRHL3* associated with NSCPO but not with NSCL/P, highlighting the discrepancy of genetic heterogeneity among various subtypes of NSOFC [11, 21]. Based on all published GWAS to date, phenotypic stratification reveals differences in the numbers of lead SNPs among the three major subtypes, with 787, 913, and 843 lead SNPs reported for NSCL/P, NSCLO, and NSCPO, respectively (Figure 3b). Candidate genes based on three subtypes of NSOFC have been summarized in Figure 3c.

In a GWAS, whether variant allele frequencies are evaluated by arrays or by sequencing [60, 61], often over 1 million tests of association between genetic variants and a phenotype are conducted, resulting in a need to correct for these many tests and exclude the false positive variants. The Bonferroni correction is commonly used but is probably exceedingly conservative because it assumes that each variant is an independent test when in fact many are in LD. Because this stringent correction will discard some true associations, variants that are near but below genome-wide significance (5e − 08 < *p*_value < 1e − 06) are often considered as potentially causative; we list such variants from multiple studies in Table S2 [5–16, 18–27, 30–32, 35, 36].

### 2.2. The Next-Generation Sequencing Studies on NSOFC

Because GWAS only score common variants, to find rare and de novo variants contributing to the heritability of NSOFC, recently, there have been at least five whole-genome sequencing (WGS) studies [62–66] and nine targeted sequencing studies focusing on NSOFC (summarized in Tables S3 and S4, respectively) [67–75]. Some functional variants within noncoding regions were found by targeted sequencing. For example, Leslie et al. found new variants through targeted sequencing of 13 GWAS-selected regions and confirmed the functional impact of noncoding variants like rs227727, disrupting the enhancer activity, and a noncoding de novo mutation affecting the enhancer activity ability of *FGFR2* through functional analysis [72]. Li et al. conducted targeted sequencing of an interval harboring *IRF6*, identifying rs12403599, an SNP within the *IRF6* promoter, as a risk factor for NSCL/P [73]. The first large-scale WGS of OFCs in parent-offspring trios was carried out by Mukhopadhyay et al. in 2020, recognizing a novel locus on Chromosome 21 as a suspected risk element for OFCs in Colombians [64]. Yu et al. identified a rare variant in *PDGFRA* (c.C2740T; p.R914W) via WGS and confirmed its biological function by zebrafish mutants [66]. Importantly, WGS of any individual will uncover thousands of variants relative to the reference genome [76]. Identifying causal variants among them is a challenge, and efforts to identify causal variants are guided by the list of known OFC-associated genes and by understanding of the gene regulatory networks governing the development of relevant orofacial tissues (neural crest and oral epithelium).

## 3. Prioritization of Noncoding Variants of NSOFC

With the advancement of GWAS and next-generation sequencing NGS, numerous genetic variants have been associated with NSOFC. However, most of these variants reside in noncoding regions, making it challenging to identify those that are truly causal and functionally relevant. Prioritization strategies have therefore become indispensable for refining large sets of associated variants into biologically meaningful candidates, reducing the complexity and cost of functional validation. To date, three major types of prioritization strategies have been widely employed in NSOFC studies—statistical fine-mapping, epigenetic fine-mapping, and machine learning–based models—each offering distinct mechanisms for highlighting variants with regulatory or pathogenic potential (comparative features summarized in Table 1). The specific applications of prioritization strategies in NSOFC-associated variant studies are detailed in Table S5 [8, 9, 11–14, 16, 18, 19, 21–25, 28, 30, 36, 44, 67–69, 72–74, 77–83, 88–92, 94–97, 102–104].

### 3.1. Statistical Fine-Mapping for Variant Prioritization

Fine-mapping uses a variety of statistical methods to examine LD structures and haplotype blocks as a way to narrow down the list of potential causal variants [109, 110]. Heuristic fine-mapping methodologies focus on assessing the LD structure surrounding a lead SNP [46], combine the lead SNP with SNPs within the same haplotype block [84], or apply conditional analysis to uncover independent signals within the region [85]. However, these approaches overlook the collective impact of SNPs and lack objective criteria for screening causal variants. Penalized regression models are aimed at enhancing stability by reducing SNP data to a smaller subset closely associated with the trait in question and operating by simultaneously estimating the effects of SNPs and shrinking coefficients towards zero [111]. Bayesian methods assume the presence of a solitary causal variant within a specific locus of interest and integrate prior probabilities concerning genetic architecture with observed GWAS data; these approaches yield posterior inclusion probabilities for each SNP [86, 112]. Ludwig et al. used a Bayesian refinement approach for each of the risk loci for NSCL/P (2p21, 8q24, 14q22, 15q24, and 19p13) identified from GWAS of the European population and revealed potential causal variants at each locus [24]. Butali et al. used a Bayesian method to fine-map the 8q24 region for NSCL/P, and by comparing samples among African, European, and Asian ancestries, they nominated a potential causal variant within this region [13]. Bayesian methods, unlike traditional those relying solely on *p*_values, enable direct comparison of posterior inclusion probabilities for SNPs, thereby enhancing the efficiency of fine-mapping. They also effectively control the influence of SNPs with larger effects by considering the collective effects of SNPs, consequently enhancing sensitivity in detecting SNPs with smaller effects. Compared to methods based solely on SNP correlation with the lead SNP, Bayesian methods tend to prioritize fewer but more likely to be causal SNPs [87]. However, as they heavily rely on prior probabilities, improperly chosen priors may introduce bias into estimations and fine-mapping conclusions.

eQTL analyses, which reveal associations between DNA variant alleles and gene expression levels, have been used to identify disease susceptibility–associated genes [47, 89]. An eQTL analysis of orbicularis oris muscle mesenchymal stem cells identified the SNP rs1063588 and the gene *MRPL53* associated with the risk of NSCL/P in a Brazilian sample [88]. Li et al. also produced an eQTL dataset from 40 lip tissues obtained from Chinese NSCL/P patients and further combined this eQTL dataset with risk SNPs from published GWAS data, revealing 243 SNPs that are associated with expression levels of 18 genes and with risk for NSCL/P [90]. The success of this approach is currently limited by a scarcity of eQTL datasets from tissues relevant to the pathogenesis of NSOFC.

### 3.2. Epigenetic Fine-Mapping for Variant Prioritization

Recent advances in analyses of chromatin accessibility and architecture permit the prioritization of DNA variants based on their presence in DNA with the epigenetic features of regulatory elements [98]. For instance, the assay for transposase-accessible chromatin using sequencing (ATAC-seq) reveals open chromatin regions more accessible to TFs; variants within these regions are more likely to regulate gene expression [93]. Moreover, chromatin immunoprecipitation sequencing (ChIP-seq) enables the mapping of regulatory elements by selectively capturing DNA fragments harboring specific posttranslational histone modifications or TF binding sites [113]. In a post-GWAS analysis, researchers used chromatin immunoprecipitation (ChIP) followed by qPCR to demonstrate that the NSOFC risk-associated allele of rs2275035 altered the binding affinity of TF KLF4 and the enhancer activity of the element containing the SNP [77]. ATAC-seq and ChIP-seq can be employed together to locate candidate regulatory elements. For instance, a set of enhancer candidates specific to zebrafish epithelial cells was identified by integrating ATAC-seq and H3K27Ac ChIP-seq from GFP-expressing periderm cells sorted from dissociated embryos [81]. Chromosome conformation capture (Hi-C) allows for efficient elucidation of chromatin interactions which reveal topologically associating domains (TADs) and enhancer–promoter interactions [99]. Xiao et al. used digestion-ligation-only Hi-C (DLO Hi-C) to identify 254 potentially functional SNPs associated with NSCPO within active enhancers of oral epithelial cells, which are physically associated with 1718 promoters in the human oral epithelial cell line [97]. A recent study showed that noncoding variants within regulatory elements can indirectly affect transcription through altering DNA methylation or modulating the three-dimensional (3D) conformation or accessibility of chromatin, and these epigenetic effects may vary in different cell types or environments, adding an additional challenge to identifying causal variants [100]. In summary, methods of mapping epigenetic features allow researchers to prioritize and interpret genetic variants based on their presence in elements likely to have a regulatory function.

### 3.3. Machine Learning Models for Assessing Noncoding Variants

Machine learning techniques have focused on identifying shared sequence features of regulatory elements, in hopes of prioritizing disease-associated variants without the need for laborious biochemical approaches. Although researchers can assess epigenetic features of chromatin and determine whether disease-associated variants lie within active regulatory elements, the methods to evaluate such features are complex, and often, the relevant tissue is difficult to access (e.g., embryonic palate tissue). Machine learning models for evaluating shared sequence features of active regulatory elements range from relatively basic logistic regression and support vector machine (SVM) to more complex models like random forests and neural networks [101, 106, 107]. Recently, Zhang et al. used 43 validated SNPs as training data and built seven models based on different machine learning methods and utilized the models to measure the accuracy of SNP risk assessment in Chinese Han populations. They found that the logistic regression model had the best predictive performance, followed by SVM [102]. The gapped k-mer support vector machine (gkmSVM), based on the enrichment weights assigned to all 10-mers within a training dataset, has been tested to predict approximately 90% of enhancer sequences revealed by the ENCODE project [105, 114]. The gkmSVM evaluation model could efficiently assess the effect of risk SNPs on tissue-specific enhancer activity, prioritizing causal variants for further biological validation experiments. Liu et al. trained three gkmSVM classifiers on sets of putative enhancers, recognized by ATAC-seq and H3K27Ac signals, from (1) zebrafish periderm cells, (2) embryonic mouse palate epithelium, and (3) a human oral epithelium cell line, respectively, to predict the effects of 14 OFC-associated SNPs near *KRT18* on enhancer activity; interestingly, all three classifiers predicted SNP (rs2070875) to have the strongest impact on enhancer activity [81]. Although gkmSVM improves prioritization of noncoding variants, the accuracy of the prediction is highly dependent on the training sets, which may include false positives. Additionally, the “black box” quality of some machine learning algorithms hinders the interpretability of their results [115].

Combined Annotation–Dependent Depletion (CADD) is a widely used machine learning model for prioritizing causal variants by predicting variants' impacts. Compared to other approaches that are trained on single annotations or on a limited set of genomic variants with known pathogenic versus benign status, CADD is trained on less biased and much larger datasets, integrating over 60 genomic features into a single measure (i.e., the *C* score) for each variant [108, 116]. Because *C* scores reflect pathogenicity, allelic diversity, functional annotations, and known risk variants, their ability to prioritize functional and pathogenic variants exceeds that of other methods [116]. The application of CADD in the study of variants associated with NSOFC is a promising area for future investigation.

## 4. Functional Validation of Noncoding Variants

**Figure 4:**
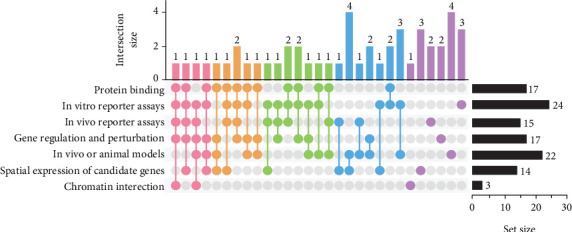
Experimental strategies for functional validation of NSOFC-associated genetic variants. Diverse experimental strategies are used to validate noncoding variants. We surveyed 48 studies and used text mining of abstracts and metadata to classify the experimental approaches used to functionally validate noncoding variants. Seven validation categories were defined: (1) protein binding, (2) in vitro reporter assays, (3) in vivo reporter assays, (4) gene regulation and perturbation, (5) in vivo or animal models, (6) spatial expression analyses of candidate genes, and (7) chromatin interaction. The UpSet plot summarizes the number of studies employing different combinations of these approaches. Each vertical bar represents the number of studies (intersection size) that used a specific combination of validation types, as indicated by the filled dots below. The color of the bars corresponds to the number of validation categories used in each combination: pink, 5; orange, 4; green, 3; blue, 2; and purple 1. Horizontal bars on the right show the total number of studies employing each individual validation approach (set size). The figure was generated in R using the UpSetR package (based on data from Table S6) and further refined in Adobe Illustrator.

Acknowledging evidence that noncoding variants may in some cases contribute to the pathogenesis of NSOFC by altering ncRNAs [43, 44], we here focus on variants which disrupt enhancers and promoters to modulate gene expression, known as regulatory variants. Using text mining, we extracted and categorized methods of functional validation commonly employed in NSOFC studies into seven major classes: (1) protein binding, including ChIP, electrophoretic mobility shift assays (EMSAs), and flanking restriction enhanced pulldown (FREP); (2) in vitro reporter assays, including luciferase assays and massively parallel reporter assays (MPRAs); (3) in vivo reporter assays, including transient transgenic enhancer assays in zebrafish and lacZ reporter assays in mice; (4) in vitro gene regulation and perturbation assays, including clustered regulatory interspaced short palindromic repeat (CRISPR)/clustered regulatory interspaced short palindromic repeat–associated protein (Cas) 9–mediated enhancer or SNP editing, allele-specific genome editing, RNA interference, siRNA-mediated knockdown, gene overexpression, and cellular phenotype assays such as migration, proliferation, and apoptosis analyses; (5) in vivo or animal models; (6) spatial expression analyses of candidate genes in craniofacial tissues, including in situ hybridization, immunohistochemistry, and immunofluorescence staining; and (7) chromatin interaction profiling, including chromosome conformation capture (e.g., 3C, 3C-qPCR, and Hi-C). We further examined how many studies employed multiple validation strategies and found that 40% of all articles utilized three or more experimental approaches (Figure 4). These findings underscore the need for multifaceted validation frameworks to establish the functional relevance of noncoding variants. The most frequently adopted validation strategies are elaborated below, while a systematic summary of all approaches employed in NSOFC-related studies is provided in Table S6 [7, 11–14, 16, 19, 21, 28, 34, 36, 44, 66, 67, 72, 73, 77, 81–83, 91, 94–97, 117–139].

### 4.1. Validation of the Regulatory Variants Within Regulatory Element Regions

#### 4.1.1. Protein Binding Assays

DNA variants situated in CREs have the potential to alter TF binding sites, either decreasing or increasing TF affinity, and thereby influencing gene expression [140]. EMSA and ChIP-seq (or ChIP-qPCR) have been used to investigate the effects of variants on the binding of transcription-associated proteins [11, 16, 19, 36, 72, 77, 83, 97, 122, 125, 126, 130, 132, 135]. For example, Rahimov et al. used EMSA to show that an NSCLO-associated SNP (rs642961, G > A) within an *IRF6* enhancer region reduces binding of the TF TFAP2A, supporting the possibility that the SNP directly affects disease risk [122]. Li et al. recently introduced a refinement to the conventional EMSA and FREP, which utilizes restriction enzyme cleavage sites adjacent to the targeted DNA sequence, thereby mitigating nonspecific binding to DNA probes [141]. A limitation of EMSA however is that protein binding is tested on naked DNA in vitro instead of in the context of chromatin. ChIP circumvents this limitation but requires antibodies specific to proteins of interest, which are sometimes unavailable. Combining DNA pulldown assays and mass spectrometry avoids the need for antibodies [137]. Single nucleotide polymorphism sequencing (SNP-seq) detects SNPs that bind regulatory proteins using Type IIS restriction enzymes which cut DNA at a fixed distance (31 bp) to one side of the binding site, regardless of the sequence there. A library of constructs is synthesized of elements containing the SNP-of-interest flanked by a Type IIS restriction enzyme site 31 bp away. The constructs are incubated in nuclear extracts of a disease-relevant cell type. If the SNP is bound by a regulatory protein, the construction will be protected from digestion. DNA sequencing and mass spectrometry identify the protected constructs and the proteins that bind them [142]. Notably, certain variants proximal to the core binding sites of TF may not disrupt the motif itself but can affect the TF's binding ability and affinity, rendering them unlikely to be predicted to disrupt binding, although protein binding assays will still reveal such effects [143, 144]. Additionally, assays performed in vitro such as EMSA and FREP lack the complicated biological environment and dynamic interactions and signaling networks between cells, tissues, and organisms. A persistent challenge in all protein binding assays is the availability of cell line models of the relevant embryonic cell type, usually embryonic oral epithelium or oral mesenchyme.

#### 4.1.2. Reporter Assays

A widely used approach to testing whether a disease-associated polymorphism is a regulatory variant is the reporter assay conducted in a disease-relevant cell type or in a model organism (such as mice or zebrafish) [145–147]. For example, researchers have found evidence that NSCL/P-associated variants are indeed regulatory through dual-luciferase assays in cell lines [11, 14, 36, 72, 77, 81, 95, 97, 122, 126, 129, 130, 135, 137]. High-throughput methods including MPRA and the self-transcribing activity regulatory region sequencing (STARR-seq) permit evaluation of thousands of variants simultaneously [83, 146, 148]. Kumari et al. conducted an MPRA to screen OFC-associated loci by cloning candidate regulatory sequences harboring SNPs into a barcoded reporter library and measuring their allele-specific activity in a fetal oral epithelial cell line (GMSM-K) [83]. MPRA enables the simultaneous, quantitative assessment of thousands of regulatory variants in a high-throughput reporter system, but its current application is limited by labor-intensive library construction, high sequencing cost, and reliance on episomal in vitro models. Recently, van Arensbergen et al. developed a method that takes advantage of the fact that both enhancers and promoters generate transcripts. They cloned fragments of the entire genome, a few hundred base pairs each, together with barcodes into a promoterless vector. Using this method, which they called survey of regulatory elements (SuREs), the authors screened 5.9 million SNPs and identified over 30,000 SNPs affecting the capacity of putative CREs in human K562 and HepG2 cell lines [149]. A limitation shared by all high-throughput reporter assays is the requirement for a disease-relevant cell line that is amenable to transfection.

While in vitro reporter assays are useful for indicating whether a disease-associated SNP is a regulatory variant, in vivo reporter assays can reveal the cell type relevant to the disease in which the enhancer is active [21, 81, 122, 129]. For example, Rahimov et al. tested whether rs642961, in an evolutionarily conserved element 9.7 kb upstream of *IRF6*, was a regulatory variant by conducting a reporter assay in cultured foreskin keratinocytes. They found that the SNP is in an enhancer active in those cells and that there was a modest difference in the risk and nonrisk alleles, although this did not reach statistical significance. In the same study, they generated transgenic mice and showed that an 876 bp element harboring rs642961 has enhancer activity in palate epithelium, which supports the candidacy of this SNP as being functional [122]. However, rs642961 is in LD with another OFC-associated SNP with strong support for being functional [83]; it is possible that both SNPs contribute to the risk associated with this haplotype. Similarly, with luciferase reporter assays in vitro, Liu et al. found two NSCL/P-associated SNPs (rs11170342 and rs2070875), both near the *KRT8* and *KRT18* genes, to be located in enhancers active in an oral epithelium cell line, and that the risk allele of rs2070875 significantly diminished its enhancer activity in these cells. In mouse reporter assays, one but not the other of the elements harboring these SNPs had enhancer activity in periderm [81]. Fisher et al. devised a transgenic reporter vector incorporating the Tol2 transposon, which was subsequently injected into zebrafish embryos revealing the regulatory potential of the noncoding sequence of interest [145]. This strategy was used to demonstrate tissue-specific enhancer activity of DNA elements containing SNPs relevant to NSCL/P [72, 77, 81, 94, 95, 97, 117, 129]. For instance, Liu et al. assessed potential allele-dependent effects of three such SNPs (rs2275035, rs4147828, and rs560426) using in vitro luciferase assays and then deployed transgenic zebrafish to test the tissue specificity of enhancers containing these SNPs. Reporter expression in F1 transgenic embryos revealed that chromatin elements containing rs2275035 or rs4147828 both have enhancer activity in craniofacial mesenchyme [77]. Of note, there has yet to be a report showing allele-dependent effects in an in vivo reporter assay, although this was attempted in transgenic mice using a safe harbor integration site [16]. Stable transgenic embryos may be necessary to reveal the modest effects of common variants on reporter expression in vivo.

### 4.2. Confirmation of the Targeted Genes and Validation of Their Biological Function

A persistent challenge in the post-GWAS era is to identify the gene that is regulated by a given functional SNP. The number of probable candidate genes is limited to those within the same TAD [150]. TAD boundaries are recognized by high levels of CTCF binding [151], and the boundaries are similar across most cell types [152]. Ultimately, confirming the gene whose expression is affected by a noncoding SNP requires engineering the genotype of the SNP in a relevant cell line and measuring expression of the genes within the TAD, starting with genes implicated in craniofacial development. Currently, to engineer precise changes to the genome, most authors use CRISPRs and Cas systems, which generate double-stranded breaks in DNA, together with a single-stranded oligonucleotide template [153, 154]. Liu et al. used this approach to engineer an oral epithelium cell line to be homozygous for either the risk or nonrisk allele of SNP rs4147828, which is located in an intron of *ABCA4* [77]. Chromatin configuration analyses revealed that the risk allele disrupts the interaction between the enhancer containing this SNP and the promoter of the adjacent *ARHGAP29* gene and reduces expression of *ARHGAP29* but not of *ABCA4*, supporting *ARHGAP29* as the targeted gene [77]. CRISPR/Cas9-mediated homology-directed repair was used to engineer the genotype of an SNP associated with cleft palate only in human induced pluripotent stem cells which were subsequently differentiated into embryonic oral epithelium; ChIP-qPCR and expression analyses showed that the SNP interferes with binding of IRF6 at an enhancer regulating expression of *IRF6* [16]. In prime editing, an engineered guide RNA capable of replacing targeted nucleotides up to 12 bases hybridizes to the targeted DNA sequence, allowing a catalytically impaired Cas9 endonuclease to specify the sites and encode the targeted base substitutions or deletions without relying on donor DNA templates or double-stranded breaks [155]. This method addresses the limitation of base editing, which is difficult to perform over eight transversion mutations, enabling us to explore more disease mechanisms.

## 5. Perspectives

**Figure 5:**
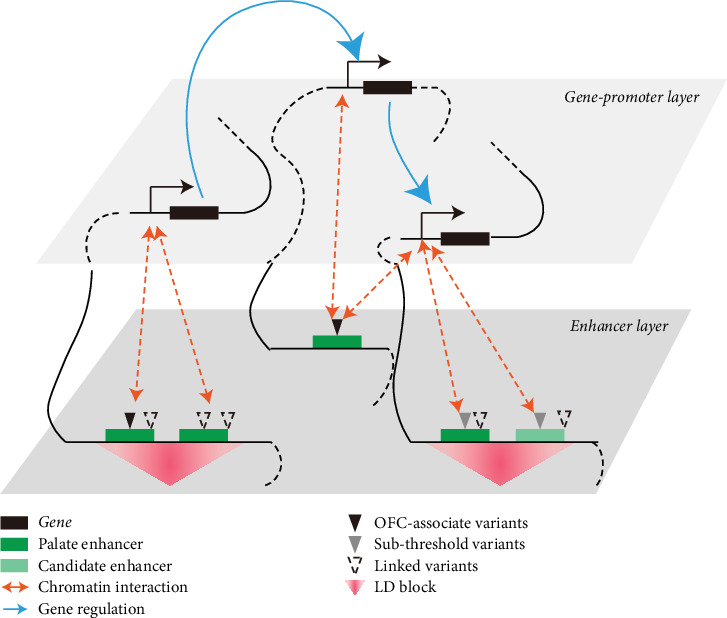
Functional annotation for potential functional variants and subthreshold SNPs. By combining palate-specific multiomics data with chromatin accessibility maps and gene regulatory networks, potential functional variants and subthreshold SNPs can be systematically annotated and mapped to their putative regulatory targets [162, 163].

Improvements in understanding the genetic underpinnings of NSOFC have come from the efforts of human geneticists searching for variants associated with risk for NSOFC and from those of developmental biologists striving to pinpoint the genes governing morphogenesis of the face. Advances in one realm have spurred them in the other. For example, *IRF6* was first identified as the gene associated with Van der Woude syndrome [156], and subsequent knockout studies in mice revealed its critical role in periderm function during palatogenesis [157, 158]. Fine-mapping and functional studies of a noncoding variant associated with OFC helped identify an enhancer region of *IRF6* that is active in embryonic oral epithelium in the mouse [122]. Conversely, *MSX1* was first recognized as being essential for palate fusion and tooth development in mice [159] and later discovered to be mutated in certain individuals with OFC or tooth agenesis [160]. Modern GWASs are aimed for ever greater scale at enhancing the generalizability of findings. For instance, recent work on gout involving a GWAS across 2.6 million individuals, including 120,295 cases, uncovered new pathogenic pathways [161]. On the developmental biology side, efforts to identify regulatory variants are benefitting from the growing availability of multiomics data, including spatial multiomics data from embryonic tissues [162, 163], which allow for precise delineation of active CREs in relevant tissues. By combining these data with chromatin interaction maps, we can directly identify the regulatory targets of noncoding variants (illustrated in Figure 5). By analyzing variants within the subthreshold range, along with palatal epigenetic data and gene regulatory networks, we can create a refined “dictionary” of OFC risk loci that will serve as an invaluable reference following genetic association studies.

Rapid advancements in artificial intelligence are set to revolutionize functional validation studies, particularly in the prioritization of candidate variants. Algorithms that integrate craniofacial epigenetic data, such as DeepFace [164], offer promising pathways to investigate the functions of OFC-associated variants and their potential regulatory mechanisms. With refined insights into the sequence features of CREs and other regulatory regions—powered by machine learning and deep learning models like the gkmSVM-based DNA scoring system [165] and Malinois-based CRE effect prediction [166, 167], and machine learning approaches to finding regulatory variants have been reviewed [168]—we can more precisely identify risk alleles that influence CRE activity or modulate target gene expression in a palatal cell-type–specific context. Recent advances have introduced tools like AlphaGenome, a deep learning model that interprets long genomic sequences to predict gene regulation and variant effects [169]. It has accurately predicted that certain noncoding mutations in leukemia indirectly activated nearby oncogenes. While powerful, AlphaGenome still struggles with predicting the effects of long-range regulatory variants and lacks training on cell-type–specific regulatory contexts. Future extensions incorporating craniofacial epigenomic datasets and cell-type–specific training regimes hold promises to adapt AlphaGenome for OFC-associated variant prediction.

Despite recent progress, several challenges remain. As most risk variants lie in noncoding regions, interpreting their function is nontrivial, given the intricate and cell-type–specific nature of gene regulation. The majority of experimental validation methods remain low-throughput and labor-intensive, and artificial intelligence approaches are still nascent, unable to accurately predict the functions of CREs under dynamic cell changes due to the fixed training datasets. Furthermore, while enhancer–promoter interactions have received the most attention, variants affecting ncRNAs—such as those disrupting miRNA binding sites, long noncoding RNA (lncRNA) function, or RNA secondary structures—also play essential roles in disease etiology but remain poorly characterized.

Finally, as most functional SNPs lie in noncoding regions, particularly in CREs, their effects are typically mediated through target gene modulation. Hence, mapping the relationships between regulatory variants and their target genes not only clarifies OFC pathogenesis but also serves as a framework to uncover pleiotropic effects. Tracing shared target genes across diseases may uncover candidate markers for comorbidities, offering new opportunities for cross-disease genetic screening.

In conclusion, identifying functional noncoding variants informs understanding of the molecular pathogenesis of OFCs. In turn, such an understanding will guide the discovery of biomarkers for diagnosis and targets for advanced therapy, ultimately improving clinical outcomes for affected individuals.

## Figures and Tables

**Table 1:** Comparison of prioritization strategies for NSOFC-associated variants.

**Category**	**Method**	**Description**	**Advantages**	**Limitations**	**Ref**	**Application**
Statistical fine-mapping	LD analysis	Identifies variants in strong LD with lead SNPs	Easy to implement; visualizes LD structure	Ignores SNP joint effects; affected by population history; arbitrary thresholds	[46]	[8, 9, 11–14, 16, 22–25, 28, 30, 36, 44, 69, 72–74, 77–83]
	Haplotype block analysis	Groups variants by LD-defined haplotype blocks	Reflects inheritance patterns	Block boundaries are arbitrary; ignores joint SNP effects	[84]	
	Conditional analysis	Detects secondary signals conditioned on lead SNPs	Identifies independent signals	High false positive rate with many tests; unstable with many SNPs	[85]	
	Bayesian refinement	Estimates posterior probabilities for causal SNPs	Less biased than threshold-based methods; quantifies uncertainty; detects weak signals	Computationally demanding; sensitive to prior choice; reduced PIP in high LD	[86, 87]	[13, 18, 24]
	eQTL integration	Links SNPs to gene expression to infer function	Provides gene-level interpretation; useful for noncoding variant prioritization	Requires relevant eQTL data; tissue-specific limitations	[47]	[19, 21, 28, 36, 67, 69, 88–92]

Epigenetic fine-mapping	ATAC-seq	Maps accessible chromatin regions	High throughput; sensitive to open regulatory elements	Requires high-quality samples; limited accessibility in clinical settings	[93]	[14, 16, 19, 24, 77, 81, 83, 91, 94–97]
	ChIP-seq	Maps histone marks or TF binding sites	Identifies active enhancers and TF targets	Cell- and condition-specific limitations; dependent on antibody quality	[98]	
	Hi-C/DLO-Hi-C	Captures 3D chromatin architecture	Maps long-range TADs and loops; reveals enhancer–promoter interaction	Low resolution; restricted by enzyme cutting; complex to analyze and interpret	[99]	
	DNA methylation profiling	Assesses CpG methylation levels to infer epigenetic regulation	Reflects stable gene regulation; captures repressive marks	Cell- and stage-specific; unclear causal direction	[100]	

Machine learning models	Logistic regression	Linear model leveraging genomic or sequence features	Easy to implement; interpretable; suitable for moderate-sized datasets	Cannot capture complex non-linear relationships; poor scalability to large datasets	[101]	[81, 97, 102–104]
	SVM/gkmSVM	Classifies data by maximizing margin in feature space (using k-mer sequence patterns)	High predictive accuracy; effective in enhancer modeling	Requires well-curated training data; black box nature hinders interpretability	[105]	
	Random forest	Tree-based ensemble learning method for classification	Handles nonlinear patterns; robust to overfitting	Less transparent; sensitive to imbalanced data	[106]	
	Neural networks	Deep learning model capturing complex data patterns	Captures nonlinear relationships; tolerant to noise; high modeling power	Requires large training datasets; computationally intensive	[107]	
	CADD	Integrates multiple annotations to score variant pathogenicity	Broadly adopted; comprehensive variant assessment; trained on unbiased large datasets	Not disease- or tissue-specific	[108]	[9, 67, 68, 72, 74, 96]

Abbreviations: Application, studies applied the corresponding method to prioritize NSOFC-associated variants; ATAC-seq, assay for transposase-accessible chromatin using sequencing; CADD, Combined Annotation–Dependent Depletion; ChIP-seq, chromatin immunoprecipitation sequencing; DLO-Hi-C, digestion-ligation-only Hi-C; eQTL, expression quantitative trait locus; gkmSVM, gapped k-mer support vector machine; Hi-C, high-throughput chromosome conformation capture; LD, linkage disequilibrium; Ref, reference; SNP, single nucleotide polymorphism; SVM, support vector machine; TAD, topologically associating domain.

## Data Availability

Data sharing is not applicable to this article as no datasets were generated or analyzed during the current study.

## Supporting Information

Supporting Information 1 Table S1. Significant genetic variants associated with NSOFC identified through GWAS. Summary of 2068 lead SNPs significantly associated with NSOFC (*p* < 5e − 08), compiled from 32 GWAS and whole-genome meta-analyses. The table includes information on SNP ID, genomic position, reference and alternative alleles, nearby gene(s), statistical significance (*p* value), ethnicity of the study population, and corresponding reference.

Supporting Information 2 Table S2. Suggestive genetic variants associated with NSOFC identified through GWAS. Summary of potentially causative variants associated with NSOFC that show suggestive associations (5e − 08 < *p* < 1e − 06), derived from 27 GWAS and meta-analyses. The table includes information on SNP ID, genomic position, reference and alternative alleles, nearby gene(s), statistical significance (*p* value), ethnicity of the study population, and corresponding reference.

Supporting Information 3 Table S3. Genetic variants associated with NSOFC identified by WGS. Summary of genetic variants associated with NSOFC identified through WGS. This table compiles data from five published WGS studies, including information on variant ID, genomic position, reference and alternative alleles, nearby gene(s), variant class, study population ethnicity, and corresponding reference.

Supporting Information 4 Table S4. Genetic variants associated with NSOFC identified by targeted sequencing. Summary of genetic variants identified in NSOFC-related genes through nine targeted sequencing studies, including information on variant ID, genomic position, reference and alternative alleles, nearby gene(s), variant class, statistical significance (*p* value), study population ethnicity, and corresponding reference.

Supporting Information 5 Table S5. Prioritization strategies for genetic variants associated with NSOFC. Overview of prioritization strategies applied in NSOFC-associated variant studies. This table summarizes how various prioritization strategies, including statistical fine-mapping, epigenetic annotation, and machine learning–based prediction models, have been utilized to refine candidate variants from GWAS and sequencing datasets. For each study, key information is provided, including the type of prioritization strategy employed, target variants or loci, and corresponding reference.

Supporting Information 6 Table S6. Functional validation of genetic variants and susceptibility genes associated with NSOFC. Overview of experimental approaches used for functional validation of NSOFC-associated variants. This table summarizes how various functional validation methods, including protein binding assays, in vitro and in vivo reporter assays, gene regulation and perturbation assays, in vivo or animal models, spatial gene expression analyses, and chromatin interaction profiling, have been utilized to functionally characterize candidate variants identified from GWAS and sequencing datasets. For each study, key information is provided, including the specific validation techniques employed, target variants or loci, and corresponding reference.
